# Fluid management in sepsis: 5 reasons why less fluid might be more rational

**DOI:** 10.62675/2965-2774.20240111-en

**Published:** 2024-08-07

**Authors:** Thiago Masashi Taniguchi, Leandro Utino Taniguchi

**Affiliations:** 1 Universidade de São Paulo Faculdade de Medicina Hospital das Clínicas São Paulo SP Brazil Intensive Care Unit, Emergency Medicine Discipline, Hospital das Clínicas, Faculdade de Medicina, Universidade de São Paulo - São Paulo (SP), Brazil.

The main physiological reason for fluid administration during hemodynamic resuscitation is not to increase blood pressure (BP) or to compensate for fluid loss. Fluid administration is all about enhancing cardiac output (CO), according to the Frank–Starling law.^( 1 )^ It is expected that during shock states, this improvement in CO might increase oxygen delivery, peripheral perfusion and, hopefully, survival. A landmark single-center trial was published by Rivers et al. in 2001 and reported a 16% absolute mortality reduction in septic patients with a hemodynamic resuscitation bundle guided by central venous saturation (early goal-directed therapy).^( 2 )^ Although this protocol was associated with approximately 13L of fluid administration in the first 72 hours after enrollment,^( 2 )^ the astonishing mortality reduction promoted its worldwide adoption (the "early goal era").

However, subsequent international multicenter trials could not replicate Rivers et al. results, and the intervention was associated with higher hospitalization costs.^( 3 )^ Further studies have suggested that early goal-directed therapy may not improve outcomes in septic patients and that outcomes may be similar between approaches that prioritize additional fluid administration and approaches that prioritizes the use of vasopressors (as recently reviewed by Zampieri et al).^( 4 )^

Although aggressive fluid administration has long been considered the cornerstone of sepsis resuscitation, an emerging body of evidence does not support this approach and suggests that it is associated with fluid overload-induced organ dysfunction.^( 5 )^ Therefore, a rational evidence-based approach should guide fluid therapy in septic patients. In the following sections, we will provide several major reasons why a liberal fluid approach should be avoided.

## 1^ST^ REASON: FLUID HEMODYNAMIC EFFECTS ARE FLEETING

After the administration of intravenous crystalloids (the most common type of fluid expansion in shock), the increase in CO does not last for more than one hour. In a prospective study conducted by Nunes et al., patients with circulatory shock received a fluid challenge of 500mL of crystalloids. Although CO peaked at 30 minutes, it progressively decreased thereafter, returning to baseline values after 60 minutes.^( 6 )^

As a consequence, after the initial fluid administration recommended by international guidelines (the famous "rapid 30mL/kg of crystalloid"),^( 7 )^ unstable patients will probably require vasoactive drugs. In a recent trial, a restrictive fluid strategy (which favored early vasopressor infusion) after initial fluid administration was not associated with a different mortality rate compared to a liberal strategy.^( 8 )^ However, this strategy might mitigate the use of short-lived therapy (fluid administration) in favor of early use of long-lived titratable efficacious therapy in sepsis-induced vasodilated hypotension.

## 2^ND^ REASON: COIN-TOSS PROBABILITY IN FLUID RESPONSIVENESS ASSESSMENT

The most common hemodynamic variable used to predict fluid responsiveness is central venous pressure (CVP).^( 9 )^ This fact is a matter of concern since it has long been demonstrated that CVP poorly predicts fluid responsiveness. Central venous pressure was not better than flipping a coin for predicting fluid responsiveness in a meta-analysis including 43 studies, which revealed a summary area under the curve value of 0.56.^( 10 )^

A multicenter study (including 311 units from 43 countries) demonstrated that 42% of fluid challenges are performed without any hemodynamic evaluation.^( 9 )^ Since only approximately 50% of patients are fluid responders,^( 10 )^ this practice of blindly giving fluids is another coin toss bet.

## 3^RD^ REASON: FLUID RESPONDERS RAPIDLY BECOME NONRESPONDERS

Even those patients whose CO increases after fluid administration (fluid responders) evolve into a fluid-unresponsive state. In a secondary analysis of the ANDROMEDA-SHOCK trial, fluid responsiveness disappeared in almost all patients during the 8-hour intervention period and after receiving a median of only 1500mL^( 11 )^ The authors also reported that fluid boluses could be stopped after nonfluid responsiveness without any negative impact on clinically relevant outcomes. Furthermore, within only 8 hours of sepsis therapy, fewer than 5% of the enrolled patients evaluated in the ANDROMEDA-SHOCK trial were fluid responders. Thus, even those who might respond to early fluid expansion rapidly become nonresponders.

## 4^TH^ REASON: MANY FLUID-RESPONSIVENESS ASSESSMENT TOOLS HAVE LIMITED APPLICABILITY IN INTENSIVE CARE UNIT PATIENTs

Since almost all patients become fluid nonresponders within a short timeframe, guidelines advocate that after the initial 30mL/kg administration, clinicians should use dynamic measures to guide fluid resuscitation.^( 7 )^ Dynamic fluid responsiveness variables, such as pulse pressure or stroke volume variation, usually have strict conditions that are rarely met in intensive care unit (ICU) patients. A prospective cohort study conducted by Mendes et al. evaluated the prevalence of ventilatory conditions for dynamic fluid responsiveness prediction. Only 2.9% of ICU patients fulfilled the conditions required for hemodynamic evaluation.^( 12 )^ Notably, passive leg-raising-based functional monitoring does not present such limitations and might be more applicable.

Recently, the emerging concept of fluid tolerance has gained attention^( 13 )^^,^ but clinical evaluation is of limited value. One might hear the following expression concerning the clinical assessment of fluid tolerance: "I can't hear lung crepitations yet, so it won't be a problem to keep the fluid expansion". This statement is inaccurate. The FEAST trial, which was conducted in resource-limited settings in Africa, compared fluid boluses with no boluses in septic children and monitored fluid administration with only clinical findings (such as vital signs, lung auscultation, peripheral perfusion, and the Cushing triad).^( 14 )^ The results revealed an increase in mortality with fluid boluses limited only by clinical evaluation of fluid tolerance in hypoperfused critically ill children.

## 5^TH^ REASON: FLUIDS ARE DRUGS WITH SERIOUS ADVERSE EFFECTS

Fluid therapy is not an innocuous treatment. It has specific hemodynamic effects that might be beneficial in low-flow states. However, as with any drug, it should be administered to those who might benefit from it (fluid responders) at the correct dose (at least the initial 30mL/kg, but more fluids are probably less efficacious), with proper monitoring. However, many clinical trials in septic patients have demonstrated that usual care is inherently associated with the administration of large volumes.^( 3 , 8 , 15 )^ Similar to any therapy in excess/unnecessary amounts, detrimental effects might accumulate, such as organ complications (such as worsening of lung function and renal failure) and worse outcomes (duration of mechanical ventilation, ICU stay, and occurrence of cardiovascular and central nervous system events).^( 5 , 15 )^

## LESS FLUID MIGHT BE MORE RATIONAL IN PATIENTS WITH SEPSIS: PRACTICAL CONSEQUENCES

**Figure 1:**
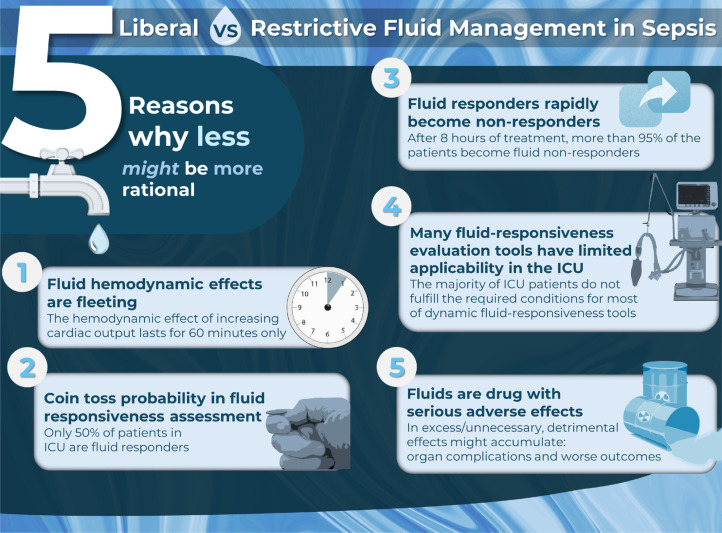
Five reasons why a restrictive fluid strategy in sepsis might be more rational.

Fluid therapy studies among septic patients have demonstrated that it is feasible, and possibly beneficial, to move from aggressive (much in excess of 60mL/kg in some studies)^( 8 )^ and obstinate fluid administration guided by unreliable assessments (such as CVP) to a more restrictive vasopressor-based strategy guided by proper fluid responsiveness tools ( Figure 1 ). Fluids are not innocuous, and the FACTT Trial and FEAST Trial revealed the consequences of liberal fluid strategies.^( 14 , 15 )^ Therefore, we suggest that, after the initial fluid bolus reaches 30mL/kg in the resuscitation phase, further fluid administration should include small boluses (e.g., 250mL) on the basis of proper volume responsiveness tools available in the optimization phase.^( 4 )^ One should also consider the early use of vasopressors to reduce hypotension-related hypoperfusion, which can be titrated for hemodynamic targets (such as lactate and capillary refill time).

ICU - intensive care unit.

Currently, there is a new mindset about fluid therapy in sepsis. This transition should be guided by the best evidence available. In the end, this rational evidence-based strategy could lead to conservative but personalized monitoring-guided fluid therapy in septic patients.
