# Supplementary material for: Fluid management in sepsis: 5 reasons why less fluid might be more rational
Source: Crit Care Sci. 2024 Aug 7;36:e20240111en. doi: 10.62675/2965-2774.20240111-en (PMC11463987; doi:10.62675/2965-2774.20240111-en)
Supplement: Translation

# Translation: Manejo de fluidos na sepse: cinco motivos pelos quais menos fluidos pode ser mais racional

**DOI:** 10.62675/2965-2774.20240111-pt

**Authors:** Thiago Masashi Taniguchi, Leandro Utino Taniguchi

**Affiliations:** 1 São Paulo SP Brasil Unidade de Terapia Intensiva, Disciplina de Emergências Clínicas, Hospital das Clínicas, Faculdade de Medicina, Universidade de São Paulo - São Paulo (SP), Brasil.

O principal motivo fisiológico para a administração de fluidos durante a ressuscitação hemodinâmica não é aumentar a pressão arterial (PA) ou compensar a perda de fluidos. A administração de fluidos tem como objetivo aumentar o débito cardíaco (DC), segundo a lei de Frank-Starling.^( 1 )^Espera-se que, durante estados de choque, essa melhora no DC possa aumentar o fornecimento de oxigênio, a perfusão periférica e, espera-se, a sobrevida. Rivers et al. publicaram um estudo de referência em um único centro em 2001 e relataram redução de 16% na mortalidade absoluta em pacientes com sepse com um pacote de ressuscitação hemodinâmica guiado pela saturação venosa central (a terapia precoce guiada por metas, conhecida pelo termo em inglês *early goal-directed therapy* ).^( 2 )^Embora esse protocolo estivesse associado à administração de aproximadamente 13L de fluido nas primeiras 72 horas após a inclusão,^( 2 )^a surpreendente redução da mortalidade promoveu sua adoção mundial (a era da terapia precoce, ou *the early goal era* ).

No entanto, estudos multicêntricos internacionais subsequentes não conseguiram replicar os resultados de Rivers et al., e a intervenção foi associada a custos mais altos de hospitalização.^( 3 )^Outros estudos sugeriram que a terapia precoce guiada por metas pode não melhorar os desfechos em pacientes com sepse, e os desfechos podem ser semelhantes entre abordagens que priorizam a administração de fluidoterapia suplementar e abordagens que priorizam o uso de vasopressores, conforme revisado recentemente por Zampieri et al.^( 4 )^

Embora a administração agressiva de fluidos tenha sido considerada por muito tempo a pedra angular da ressuscitação na sepse, um conjunto emergente de evidências não apoia essa abordagem e sugere que ela está associada à disfunção orgânica induzida pela sobrecarga de fluidos.^( 5 )^Desse modo, uma abordagem racional fundamentada em evidências deve orientar a fluidoterapia em pacientes com sepse. Nas próximas seções, apresentamos vários motivos importantes pelos quais uma abordagem liberal de fluidos deve ser evitada.

## PRIMEIRO MOTIVO: OS EFEITOS HEMODINÂMICOS DOS FLUIDOS SÃO EFÊMEROS

Após a administração de cristaloides intravenosos (o tipo mais comum de fluido de expansão no choque), o aumento do DC não dura mais de 1 hora. Nunes et al. conduziram um estudo prospectivo em que pacientes com choque circulatório receberam uma prova de volume de 500mL de cristaloides. Embora o DC tenha atingido o pico aos 30 minutos, ele diminuiu progressivamente depois disso, retornando aos valores basais após 60 minutos.^( 6 )^

Como consequência, após a administração inicial de fluidos recomendada pelas diretrizes internacionais (os famosos "30mL/kg de cristaloides rápidos"),^( 7 )^os pacientes instáveis provavelmente precisarão de drogas vasoativas. Em um estudo recente, uma estratégia restritiva de fluidos (que favoreceu a infusão precoce de vasopressores) após a administração inicial de fluidos não foi associada a uma taxa de mortalidade diferente da estratégia liberal.^( 8 )^No entanto, essa estratégia pode mitigar o uso de terapia de curta duração (administração de fluidos) em favor do uso precoce de terapia eficaz titulável de longa duração na hipotensão vasodilatada induzida por sepse.

## SEGUNDO MOTIVO: PROBABILIDADE DE CARA OU COROA NA AVALIAÇÃO DA RESPONSIVIDADE A FLUIDOS

A variável hemodinâmica mais comumente usada para prever a responsividade a fluidos é a pressão venosa central (PVC).^( 9 )^Esse fato é preocupante, pois, há muito tempo, foi demonstrado que a PVC prevê erroneamente a responsividade a fluidos. A PVC não foi melhor do que jogar cara ou coroa para prever a responsividade a fluidos em uma metanálise que incluiu 43 estudos, que revelou uma área sob o valor da curva de 0,56.^( 10 )^

Um estudo multicêntrico (que incluiu 311 unidades de 43 países) demonstrou que 42% dos estímulos de fluidos são realizados sem nenhuma avaliação hemodinâmica.^( 9 )^Como apenas aproximadamente 50% dos pacientes são responsivos a fluidos,^( 10 )^essa prática de administrar fluidos às cegas é outra aposta de cara ou coroa.

## TERCEIRO MOTIVO: OS RESPONSIVOS A FLUIDOS RAPIDAMENTE SE TORNAM NÃO RESPONSIVOS

Mesmo aqueles pacientes cujo DC aumenta após a administração de fluidos (responsivos a fluidos) evoluem para um estado não responsivo a fluidos. Em uma análise secundária do estudo ANDROMEDA-SHOCK, a responsividade a fluidos desapareceu em quase todos os pacientes durante o período de intervenção de 8 horas e após receberem uma mediana de apenas 1.500mL.^( 11 )^Os autores também relataram que os bólus de fluidos poderiam ser interrompidos após a não responsividade a fluidos sem nenhum impacto negativo nos desfechos clinicamente relevantes. Além disso, em apenas 8 horas de tratamento da sepse, menos de 5% dos pacientes incluídos e avaliados no estudo ANDROMEDA-SHOCK foram responsivos à administração de fluidos. Assim, mesmo aqueles que poderiam ser responsivos à expansão precoce de fluidos rapidamente se tornam não responsivos.

## QUARTO MOTIVO: MUITAS FERRAMENTAS DE AVALIAÇÃO DA RESPONSIVIDADE A FLUIDOS TÊM APLICABILIDADE LIMITADA EM PACIENTES DE UNIDADES DE TERAPIA INTENSIVA

Como quase todos os pacientes se tornam não responsivos a fluidos em um curto período de tempo, as diretrizes defendem que, após a administração inicial de 30mL/kg, os médicos devem usar medidas dinâmicas para orientar a ressuscitação por fluidos.^( 7 )^As variáveis dinâmicas de responsividade a fluidos, como a pressão de pulso ou a variação do volume sistólico, geralmente têm condições rígidas que raramente são atendidas em pacientes de unidades de terapia intensiva (UTI). Mendes et al., em um estudo de coorte prospectiva, avaliaram a prevalência de condições ventilatórias para a previsão da responsividade dinâmica a fluidos. Apenas 2,9% dos pacientes de UTI preencheram as condições necessárias para a avaliação hemodinâmica.^( 12 )^Notavelmente, o monitoramento funcional baseado na elevação passiva das pernas não apresenta essas limitações e pode ser mais aplicável.

Recentemente, o conceito emergente de tolerância a fluidos ganhou atenção,^( 13 )^mas a avaliação clínica é de valor limitado. É possível ouvir a seguinte expressão com relação à avaliação clínica de tolerância a fluidos: "Ainda não consigo ouvir as crepitações pulmonares, portanto, não será problemático manter a expansão de fluidos". Essa afirmação é imprecisa. O estudo FEAST, realizado em ambientes com recursos limitados na África, comparou a administração de bólus de fluidos com a não administração de bólus em crianças com sepse e monitorou a administração de fluidos apenas com achados clínicos (como sinais vitais, ausculta pulmonar, perfusão periférica e a tríade de Cushing).^( 14 )^Os resultados revelaram aumento na mortalidade com bólus de fluidos limitados apenas pela avaliação clínica de tolerância a fluidos em crianças em estado crítico hipoperfundidas.

## QUINTO MOTIVO: OS FLUIDOS SÃO MEDICAMENTOS COM EFEITOS ADVERSOS GRAVES

A fluidoterapia não é um tratamento inócuo. Ela tem efeitos hemodinâmicos específicos que podem ser benéficos em estados de baixo fluxo. No entanto, como acontece com qualquer medicamento, deve ser administrado àqueles que podem se beneficiar dele (responsivos a fluidos) na dose correta (pelo menos os 30mL/kg iniciais, mas um volume maior de fluidos provavelmente é menos eficaz), com monitoramento adequado. No entanto, muitos estudos clínicos em pacientes com sepse demonstraram que os cuidados habituais estão inerentemente associados à administração de grandes volumes.^( 3 , 8 , 15 )^Da mesma forma que qualquer terapia em excesso/quantidades desnecessárias, efeitos deletérios podem se acumular, como complicações orgânicas (como piora da função pulmonar e insuficiência renal) e desfechos desfavoráveis (duração da ventilação mecânica, internação na UTI e ocorrência de eventos cardiovasculares e do sistema nervoso central).^( 5 , 15 )^

## MENOS FLUIDO PODE SER MAIS RACIONAL EM PACIENTES COM SEPSE: CONSEQUÊNCIAS PRÁTICAS

Estudos de fluidoterapia em pacientes com sepse demonstraram que é viável e, possivelmente, benéfico passar de uma administração de fluidos agressiva e obstinada (muito acima de 60mL/kg em alguns estudos),^( 8 )^orientada por avaliações não confiáveis (como a PVC), para uma estratégia mais restritiva baseada em vasopressores, orientada por ferramentas adequadas de responsividade a fluidos ( Figura 1 ). Os fluidos não são inócuos, e os estudos FACTT e FEAST revelaram as consequências de estratégias liberais de fluidos.^( 14 , 15 )^Portanto, sugerimos que, depois que o bólus inicial de fluidos atingir 30mL/kg na fase de ressuscitação, a administração complementar de fluidos deve incluir pequenos bólus (por exemplo: 250mL) de acordo com as ferramentas adequadas de responsividade a volume disponíveis na fase de otimização.^( 4 )^Deve-se considerar também o uso precoce de vasopressores para reduzir a hipoperfusão relacionada à hipotensão, que pode ser titulada para metas hemodinâmicas (como lactato e tempo de reenchimento capilar).

**Figura 1:** Cinco motivos pelos quais uma estratégia restritiva de fluidos na sepse pode ser mais racional.

UTI - unidade de terapia intensiva.

Atualmente, há uma nova mentalidade sobre a fluidoterapia na sepse. Essa transição deve ser orientada pelas melhores evidências disponíveis. No fim, essa estratégia racional embasada em evidências poderia levar a uma fluidoterapia conservadora, mas personalizada, guiada por monitoramento em pacientes com sepse.
